# Heritable Epigenetic Variation among Maize Inbreds

**DOI:** 10.1371/journal.pgen.1002372

**Published:** 2011-11-17

**Authors:** Steve R. Eichten, Ruth A. Swanson-Wagner, James C. Schnable, Amanda J. Waters, Peter J. Hermanson, Sanzhen Liu, Cheng-Ting Yeh, Yi Jia, Karla Gendler, Michael Freeling, Patrick S. Schnable, Matthew W. Vaughn, Nathan M. Springer

**Affiliations:** 1 Microbial and Plant Genomics Institute, Department of Plant Biology, University of Minnesota, Saint Paul, Minnesota, United States of America; 2 Department of Plant and Microbial Biology, University of California Berkeley, Berkeley, California, United States of America; 3 Iowa State University, Ames, Iowa, United States of America; 4 Texas Advanced Computing Center, University of Texas–Austin, Austin, Texas, United States of America; The University of North Carolina at Chapel Hill, United States of America

## Abstract

Epigenetic variation describes heritable differences that are not attributable to changes in DNA sequence. There is the potential for pure epigenetic variation that occurs in the absence of any genetic change or for more complex situations that involve both genetic and epigenetic differences. Methylation of cytosine residues provides one mechanism for the inheritance of epigenetic information. A genome-wide profiling of DNA methylation in two different genotypes of *Zea mays (ssp. mays)*, an organism with a complex genome of interspersed genes and repetitive elements, allowed the identification and characterization of examples of natural epigenetic variation. The distribution of DNA methylation was profiled using immunoprecipitation of methylated DNA followed by hybridization to a high-density tiling microarray. The comparison of the DNA methylation levels in the two genotypes, B73 and Mo17, allowed for the identification of approximately 700 differentially methylated regions (DMRs). Several of these DMRs occur in genomic regions that are apparently identical by descent in B73 and Mo17 suggesting that they may be examples of pure epigenetic variation. The methylation levels of the DMRs were further studied in a panel of near-isogenic lines to evaluate the stable inheritance of the methylation levels and to assess the contribution of *cis*- and *trans*- acting information to natural epigenetic variation. The majority of DMRs that occur in genomic regions without genetic variation are controlled by *cis*-acting differences and exhibit relatively stable inheritance. This study provides evidence for naturally occurring epigenetic variation in maize, including examples of pure epigenetic variation that is not conditioned by genetic differences. The epigenetic differences are variable within maize populations and exhibit relatively stable trans-generational inheritance. The detected examples of epigenetic variation, including some without tightly linked genetic variation, may contribute to complex trait variation.

## Author Summary

Heritable variation within a species provides the basis for natural and artificial selection. A substantial portion of heritable variation is based on alterations in DNA sequence among individuals and is termed genetic variation. There is also evidence for epigenetic variation, which refers to heritable differences that are not caused by DNA sequence changes. Methylation of cytosine residues provides one molecular mechanism for epigenetic variation in many eukaryotic species. The genome-wide distribution of DNA methylation was assessed in two different inbred genotypes of maize to identify differentially methylated regions that may contribute to epigenetic variation. There are hundreds of genomic regions that have differences in DNA methylation levels in these two different genotypes, including methylation differences in regions without genetic variation. By studying the inheritance of the differential methylation in near-isogenic progeny of the two inbred lines, it is possible to demonstrate relatively stable inheritance of epigenetic variation, even in the absence of DNA sequence changes. The epigenetic variation among individuals of the same species may provide important contributions to phenotypic variation within a species even in the absence of genetic differences.

## Introduction

Much of the heritable variation within a species is a consequence of differences in the primary DNA sequence of different individuals. However, there is growing evidence for heritable variation in the absence of DNA sequence polymorphisms, termed epigenetic variation [1]. Cytosine methylation is one of the molecular mechanisms that can contribute to epigenetic variation and often acts to suppress the activity of transposable elements, repetitive sequences, pseudogenes, and in some cases otherwise active genes [2], [3]. There is evidence that epigenetic changes can lead to stable phenotypic variation in plant and animal species [4]–[10]. However, the abundance and role of epigenetic, relative to genetic, variation has not been well characterized. Maize (*Zea mays*) provides a useful model to study the role of epigenetic variation. Genetically, maize is a highly diverse species [11], [12] with a large, complex genome with many interspersed genic and repetitive regions [13], [14]. While in the past this complex genomic structure has complicated the ability to perform genome-wide analyses it also is likely to contribute to higher levels of epigenetic variation relative to less complex genomes such as Arabidopsis [3], [15]. In addition, there are outstanding resources for the analysis of quantitative trait variation in maize [16], [17] that may allow for a better understanding of the relative roles of genetic and epigenetic variation in controlling quantitative trait variation.

In plants, the majority of genome-wide methylation studies have been conducted in Arabidopsis [18]–[20], [8]. In these studies DNA methylation was frequently associated with heterochromatic regions, transposable elements, and repetitive DNA [18]. In general, lower levels of methylation occur within gene promoter sequences; however when present, promoter methylation shows a negative correlation with gene expression [19]. Within gene bodies, regions of DNA methylation have been observed uniquely in the CG context, but no major impact on gene expression is associated with this modification [20]. The exact role of gene body methylation is unclear, but it may preferentially affect moderately-transcribed genes [19], and be under different regulatory control than that of transposable element methylation [21]. Similar genome-wide patterns of DNA methylation have also been observed in rice and poplar [22]. A recent analysis of DNA methylation in maize used a 0.3× coverage sequencing of McrBC digested DNA to show evidence for mutually exclusive patterns of DNA methylation and H3K27me3 near genes with low, or no expression [23].

DNA methylation has been proposed to play a role in generating variation that could provide adaptation to environmental stresses [5], [24]–[27]. Two groups have recently developed epiRIL populations in which epigenetic states were altered by passage through DNA methylation mutants [28]–[31]]. The existence of quantitative trait variation in these populations suggests that alteration of DNA methylation patterns can induce phenotypic change although it is difficult to rule out the potential for primary sequence changes due to activated transposition. These studies have been very useful for documenting an important role for DNA methylation in regulating complex traits but do not provide information on natural variation for epigenetics states. There is evidence that DNA methylation patterns at specific loci can vary within Arabidopsis ecotypes [8], [32]–[35] and there are several specific examples of epigenetic variation that result in phenotypic variation in a variety of species [36]–[44]. However, there are limited analyses of genome-wide methylation variation conducted in plant species. A detailed contrast of chromosome 4 methylation patterns in *Arabidopsis thaliana* ecotypes shows very similar targeting of transposable elements and repetitive sequences, yet genic partial methylation states were highly polymorphic across ecotypes [8]. A study in maize found evidence for variable effects of CHG methylation on transcription patterns in different inbreds of maize [45].

Despite the evidence for variation in DNA methylation patterns among individuals of the same species relatively little is known about the nature of the inheritance of these methylation differences. One study in Arabidopsis found that gene body methylation was only partially heritable and was lost at a relatively high frequency [8]. Richards [5] provided a description of how methylation variation may be dependent upon, conditioned by, or independent of DNA sequence change and termed these as examples of obligatory, facilitated or pure epialleles, respectively. Obligatory epialleles exhibit different levels of DNA methylation but are entirely dependent upon DNA sequence changes at linked or unlinked sites. In contrast, facilitated and pure epialleles exhibit stochastic variation that can be conditioned with or without genetic differences, respectively. In addition, there is evidence that genetic variation at unlinked genomic regions can direct epigenetic changes elsewhere in the genome, presumably through an RNA-direct DNA methylation (RdDM) pathway [36], [46], [47].

We sought to characterize the variation in DNA methylation patterns in two maize inbred genotypes, B73 and Mo17. Genome-wide profiling of DNA methylation patterns was used to assess the relationship of methylation to chromosomal and gene structures. Although the majority of the genome shows highly similar methylation patterns in both inbreds there are several hundred differentially methylation regions (DMRs) found throughout the maize genome. The analyses of several identical-by-descent regions of the B73 and Mo17 genomes provides evidence that epigenetic variation can occur in the absence of nearby genetic polymorphisms. A population of near-isogenic lines was used to further characterize the heritable behavior of the DMRs and to assess the genomic regions that controlled the differential methylation.

## Results

**Figure 1:**
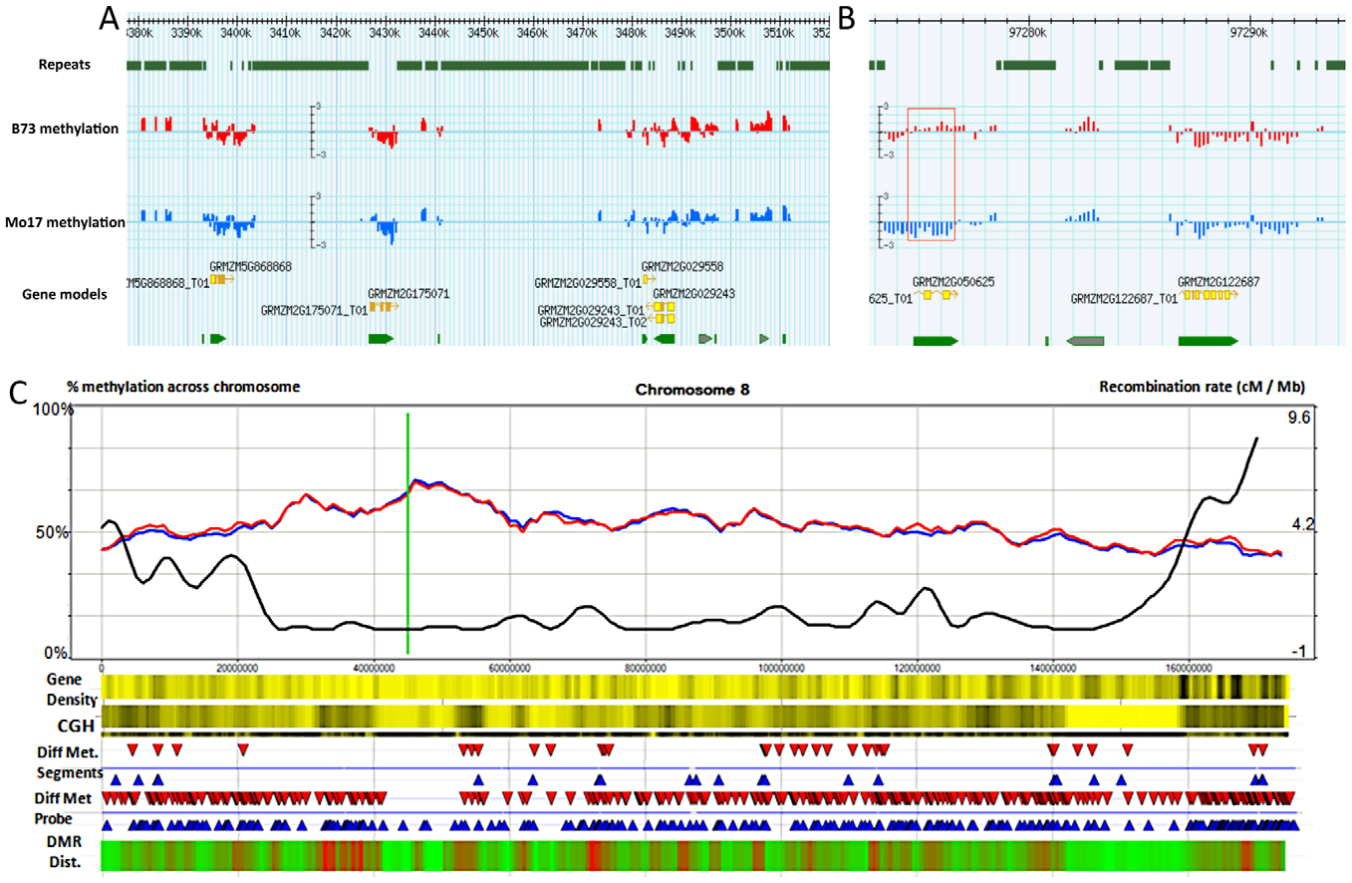
Synopsis of chromosome-level methylation and variation. (A–B) A Gbrowse view is presented for 140 kb of chromosome 8 (3,380 kb–3,520 kb) and a closer view of 22 kb (97,273–97,294K) that includes a differentially methylated region (red box). The top track shows the regions that are annotated as repetitive sequences using the MIPS/Recat repeat catalog. The next two tracks show the B73 (red) and Mo17 (blue) relative methylation levels for each of the probes within these regions. Methylation levels are defined as the normalized log2 ratio of IP enriched sample to un-enriched genomic DNA on a scale of −3 to 3 indicating methylation enrichment (>0) and depletion (<0) respectively. The bottom tracks illustrate the gene models. The gray arrows indicate gene models that were rejected from the FGS. (C) Shows a chromosomal view of methylation levels in B73 and Mo17. The percentage of methylation is plotted as a 5 Mb window sliding 1 Mb downstream across the chromosome Blue and red lines indicate percent methylation of all probes for B73 and Mo17 respectively. The green line indicates centromere position. The black line shows the cm/Mb across the chromosome. The first heatmap provides a visualization of gene density. Yellow and black values indicate lower and higher relative gene density values respectively. The second heatmap provides visualization for the genomic structural variation between B73 and Mo17 using Comparative Genomic Hybridization (CGH) values. A 5 Mb sliding window across the chromosome indicates regions of high diversity (black) to low diversity (yellow). The differential methylation regions (DMRs) are shown using red (Mo17 hypermethylation) and blue (B73 hypermethylation) arrows. The next track shows the location of individual probes that have significant (q<0.001) methylation variation between B73 and Mo17. The final heat map indicates the relative enrichment for differentially methylation probes across the chromosome with enriched regions indicated by red and regions with depleted levels of methylation variation in green.

An array platform containing 2.1 million long oligonucleotide probes was designed to profile genomic DNA methylation patterns in low-copy sequences throughout the maize genome (Table S1; Methods). Methylated DNA immunoprecipitation (meDIP) was performed on fragmented genomic DNA using a 5-methylcytosine antibody. This approach is very useful for providing cost-effective quantitative estimates of DNA methylation density [19]. This method can detect substantial differences in the proportion of methylated cytosines in genomic regions but cannot accurately assess individual bases or differentiate the different types of DNA methylation such as CG, CHG, CHH. The enrichment of methylated DNA was confirmed (Figure S1A) by assessing the enrichment for a region known to be methylated and lack of enrichment for a region known to lack significant DNA methylation [48]. meDIP was performed on three biological replicates of leaf blade tissue isolated from the third expanded leaf of greenhouse-grown B73 and Mo17 seedlings; the resulting enriched fractions were labeled and hybridized to the array along with un-enriched control input DNA. Linear model ANOVA was used to estimate values for input DNA, B73 methylation, Mo17 methylation and relative methylation in B73 and Mo17 (Figure 1).

The probe sequences were designed based on the sequence of the B73 reference genome, but previous studies have documented abundant DNA sequence polymorphisms [49] and structural variants between B73 and Mo17 [50], [51]. We investigated the methylation levels at sequences that exhibit structural genomic variation such as copy number variation (CNV) and presence-absence variation (PAV). Comparative genomic hybridization (CGH) data were obtained from the hybridization of input B73 and Mo17 genomic DNA. DNAcopy [52] was performed, followed by expectation maximization [53] model analysis to identify segments that exhibit significantly more copies in Mo17 than in B73 (M>B CNV) and to identify segments that exhibit significant fewer or no copies in Mo17 relative to B73 (M<B CNV and PAV) (Table S2). While there are examples of CNV or PAV that show high levels of methylation, there is little evidence for substantial differences in the overall methylation levels of sequences that exhibit structural genome variation relative to sequences that do not show structural variation (Figure S2, Figure S3A–S3C). Even so, for subsequent analyses, we focused on regions that do not have any evidence for CNV/PAV based on the CGH data. The values from the remaining probes were adjusted using the B73-input vs. Mo17-input hybridization coefficient to control for differential hybridization efficiency while still estimating methylation differences.

Microarray probes were selected to target low-copy regions of the maize genome by using repeat-masked sequences (provided by J. Stein and D. Ware). This repeat masking does not, however, remove all multi-copy sequences. The number of exact (100% identity and coverage) or close matches (>90% identity and coverage) was determined for each probe (Table S3). Slightly over half (58%) of the probes present on the array have only a single perfect match in the B73 reference genome and no other close matches. As the numbers of perfect or close matches for probes increase there is a significant increase in the levels of methylation they detect (Figure S3D–S3E). This copy-number dependent increase in methylation levels is observed in both B73 and Mo17 (data not shown). The subsequent genome-wide analyses of DNA methylation are confined to the subset of probes that are present as a single copy within the B73 genome (Table 1). The genome-wide analysis of methylation levels in Mo17 is further restricted to those probes that do not exhibit evidence for substantial differences in CGH values in the two inbreds (Table 1). By focusing on these subsets of probes the effects of probe copy number and genomic polymorphism on the detected methylation levels are minimized.

**Table 1:** Methylation levels in subsets of probes.

Data Set	Probes	% Probes	% methylated (50%pp)
B73_methylation	2,120,701.00	100.00	50.11
B73_methylation_unique∧ *	1,202,553.00	56.71	52.49
Mo17_methylation∼	1,940,644.00	91.51	41.37
Mo17_methylation_unique∧ * ∼	1,088,820.00	51.34	49.11

Filters (completed in order indicated above)

∧ = every third chromosome 9 probe for similar spacing relative to other chromosomes.

* = unique probes (only one perfect match in genome).

∼ = CGH filter (only probes with CGH values >−1).

The distribution of per-probe methylation estimates provides evidence for a bi-modal distribution (Figure S1B) with the two distributions accounting for methylated and un-methylated genomic regions. Application of expectation maximization allows classification of the methylation status of each probe (Table 1). The genomic distribution of DNA methylation patterns was visualized across each of the maize chromosomes (Figure 1C and Figure S4). Similar to other species, methylation levels are higher in the pericentromic regions of maize chromosomes than at the ends of chromosomal arms. There are several regions of higher methylation throughout the chromosome that do not correlate with the centromeric position and do not correlate well with cytologically visible features such as knobs or rDNA sites. In general, the relative levels of DNA methylation are inversely correlated with gene density. The relative levels of DNA methylation in parental lines also show a negative correlation with recombination rates measured in a set of intermated B73×Mo17 RILs [54]. However, the exact parents for this population may have slight differences relative to the B73 and Mo17 profiled in this study and we have not measured actual DNA methylation profiles in any specific RIL genotype.

### Comparative genomic analysis of DNA methylation dynamics for maize genes

**Figure 2:**
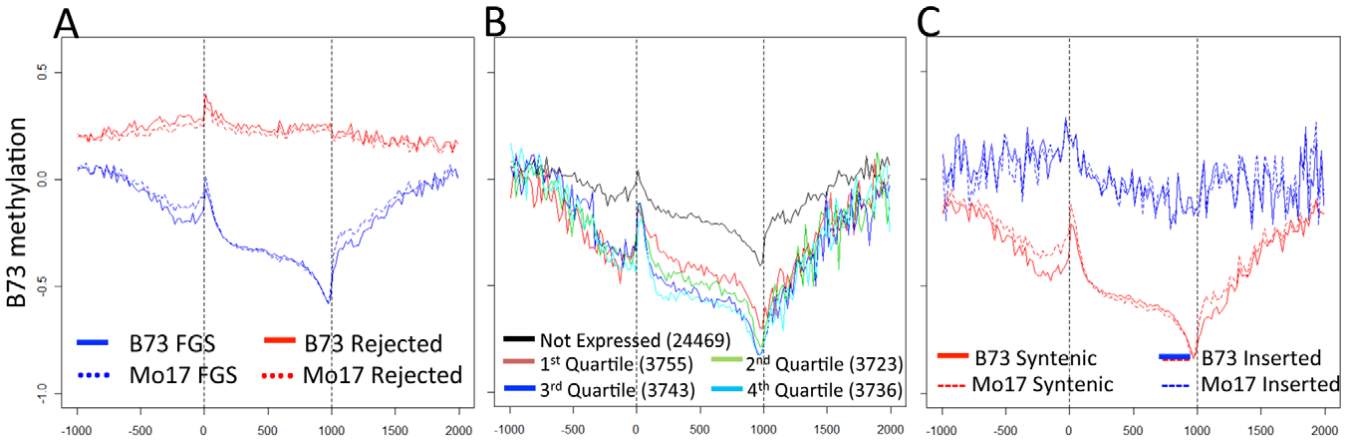
Gene body methylation and expression levels in maize genes. (A) The relative methylation levels (log2(IP/input)) were assessed for probes within 1000 bp of the transcription start and termination site. The relative position for probes within the gene was normalized to a scale of 1000. The genes in the filtered gene set (FGS) exhibit a lower methylation and a more dynamic pattern across the length of the gene than the rejected genes. The vertical dashed lines indicated the beginning and end of transcription for each gene. (B) The relative expression level for all FGS genes was assessed using published RNAseq data from B73 leaf tissue [56] and genes were assigned as not expressed or quartile 1–4 based on their expression level. In general, the genes show similar patterns of methylation but the higher expressed genes exhibit lower levels of methylation within and around the gene. (C) Each of the FGS genes was also classified according to whether it was located in a syntenic position relative to sorghum and/or rice or in a non-syntenic position. The syntenic genes exhibit much lower levels of methylation than the non-syntenic genes. This difference between syntenic and non-syntenic genes can also be seen in the regions immediately surrounding the gene.

The location of each probe was determined relative to the gene models of annotation 5a.59 (www.maizesequence.org). Version 5a.59 of the maize working gene set contains 104,369 annotated genes which include 39,384 genes models that are part of the high-confidence filtered gene set (FGS) and another 64,985 genes that were rejected from the FGS. In both B73 and Mo17 the FGS genes show substantially lower methylation levels within and surrounding the genes relative to the rejected genes (Figure 2A). The reasons for rejecting genes from the FGS include low confidence FGENESH models, probable transposons and probable pseudogenes. Rejected genes that fall into these classes exhibit significantly higher methylation than the genes in the FGS (Figure S5). The methylation pattern for FGS genes has reduced methylation in the 300 bp upstream of the transcription start site (TSS) then has a short “peak” of methylation in the very beginning of the gene which drops off quickly in the 3′ direction. There is a region of low methylation at the 3′ ends of FGS genes, but methylation returns to genome-wide average levels within 500 bp of the transcription termination site. This distribution of methylation levels, particularly the increased methylation at the 5′ ends of genes, is distinct from patterns observed in other species [55], [22] but is consistent with a previous report from maize [23]. The methylation pattern observed across the gene body is related to the distribution of CpG dinucleotides (Figure S6A–S6B). However, the analysis of the region immediately upstream of the transcription site reveals that this region with increased CpG content does not show increased DNA methylation levels which confirms hypomethylation of these promoter regions and provides evidence that observed methylation levels are not strictly driven by CpG content (Figure S6C). In addition to the dynamics of methylation along the length of the genes, there are also significant differences in the methylation levels of exons, introns and UTRs relative to intergenic probes (Figure S6D). Introns show relatively low methylation levels throughout the gene body while exon sequences exhibit relatively high methylation in the 5′ end and low methylation in the 3′ end of the gene (Figure S6E). Many of these differences reflect the relatively high CpG content of the first exons of maize genes.

To determine the relationship between DNA methylation and gene expression, the relative expression levels of FGS genes in B73 leaf tissue were used to divide genes into five categories: non-expressed; and four quartiles based on RNAseq data from Li et al. [56]. As expected, highly expressed genes show the lowest levels of methylation. There are significant differences in DNA methylation values among all quartiles of genes except between the two quartiles containing highly expressed genes (Figure S7A). Genes that are not expressed have higher levels of methylation in nearby regions as well as within the gene body (Figure 2B). We proceeded to assess methylation levels of FGS genes in a comparative genomics context. Schnable et al. [57] used comparative genomic approaches to identify homoeologous regions of the maize genome derived from a whole genome duplication event and to then assign them to sub-genome 1 and sub-genome 2 based on the level of fractionation observed. Sub-genome 1 has retained a larger proportion of the ancestral genes and generally exhibits higher mRNA expression levels as compared to sub-genome 2. Despite the trend for lower expression levels for genes in sub-genome 2 [57], there was no evidence for differences in methylation levels in genes present in sub-genome 1 relative to sub-genome 2 (Figure S7B). However, there was evidence for substantial differences in the methylation levels of genes in the FGS that are in syntenic positions relative to sorghum and/or rice relative to FGS genes that are located in non-syntenic positions (Figure 2C). The non-syntenic genes are enriched (chi-square p value<0.001) for genes that are not expressed or are in the lowest quartile of expressed genes based on the data of Li and coworkers [56].

### Variability for B73-Mo17 methylation

**Figure 3:**
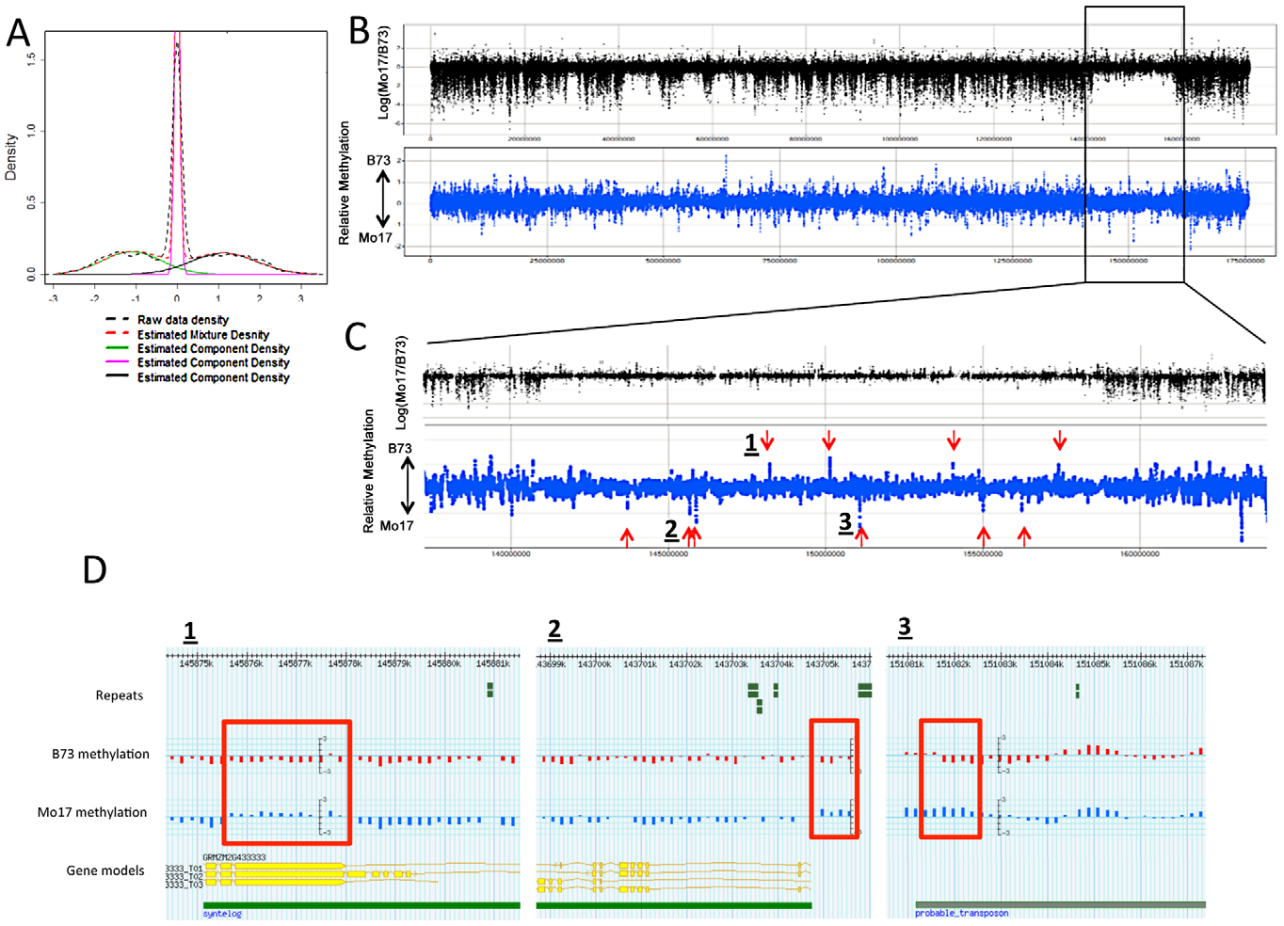
DMRs in B73 and Mo17. (A) The relative methylation levels for all probes were used to perform DNAcopy segmentation followed by expectation maximization. The black dashed lines show the observed distribution of the segment means. This distribution can be approximated (red dashed line) by a model that is derived from three normal distributions including B73 hypermethylation (right peak), Mo17 hypermethylation (left peak) and unchanged regions (middle peak). (B) The structural variation across chromosome 8 is shown in the plot with black spots. The blue spots show relative methylation in B73 and Mo17. The region of low structural diversity (boxed region) is magnified in (C). (D) Gbrowse views for three DMRs located within this region are shown. The data tracks show the position of repeats, genes, B73 methylation, Mo17 methylation and relative methylation. Each bar showing methylation values represents an individual probe. The actual DMRs are shown by the red boxes.

A visual analysis of the B73 and Mo17 methylation patterns revealed that while the majority of loci exhibit very similar patterns, there are examples of altered methylation levels between the two genotypes (Figure 1). Two different approaches were used to discover differentially methylated regions (DMRs) in B73 and Mo17. One approach identified individual probes that exhibit significant (q<0.001) differences in the contrast of B73 and Mo17 methylation (Table 2). A second approach implemented the DNAcopy segmentation algorithm on the relative methylation values followed by expectation maximization to identify segments of at least three probes that exhibit altered methylation (Figure 3A; Table 3). The per-probe analysis is capable of identifying small regions with altered methylation whereas the analysis of segments defined by adjacent probes will identify larger, high-confidence DMRs.

**Table 2:** Probes with variable DNA methylation in B73 and Mo17.

Probe Class	# Probes	Mean B73 Methylation (log_2_(meDIP/input))	Mean Mo17 Methylation (log_2_(meDIP/input))	% present in segments	% Syntenic	% Intergenic
Mo17 hypermethylation	5367	−1.15	0.81	20.4	51.6	67.4
B73 hypermethylation	4172	1.05	−1.03	23.2	37.5	72.3
All probes	1088820	−0.21	−0.21	NA	56.2	54.2

**Table 3:** Segments with variable DNA methylation levels in B73 and Mo17.

Segment Class	# Segments	Segment Mean	Avg # Probes	Avg Seg. Length	Avg # of FGS Genes	Avg # of WGS Genes	#Segs with at least 1 FGS	#Segs with at least 1 WGS	% of probes q<0.0001
Mo17 hypermethylation	402	−1.6125	5.6	1241	0.119	0.303	46	111	39.9%
No change	919	0.0010	1149.9	2148870	39.410	96.210	877	910	NA
B73 hypermethylation	288	1.7846	4.9	1219	0.087	0.285	25	74	44.4%
Unclassified	269	−0.1459	92.9	246008	3.000	8.530	197	238	NA

The DMRs identified by segmentation were further characterized as they have evidence for altered methylation from multiple adjacent probes encompassing a region of at least several hundred base pairs. There are nearly 700 DMR segments each that exhibit either B73 or Mo17 hypermethylation (Table 3; full list in table S4). The Mo17 hypermethylation segments include a total of 500 kb of DNA while the B73 hypermethylation segments include a total of 350 kb of DNA. The majority of the DMRs (674/690) are less than 5 kb in length and only one segment is over 10 kb (Figure S8A). The majority of the DMRs occurred in intergenic regions and relatively few even overlap with a FGS gene or a member of the WGS (Table 3). Those genes that were contained within DMRs were enriched for non-syntenic genes, inclusion in sub-genome 1, and for those that are not expressed in leaf tissue (Figure S8B–S8C). A genomic visualization of probes and/or segments of differential methylation (Figure 1; Figure S4) revealed a non-uniform genomic distribution.

The DMRs may be conditioned by local sequence differences in B73 and Mo17 or may be the result in stochastic epigenetic differences that are not directly attributable to genetic differences. We focused on genomic regions of low diversity to identify potential examples of epigenetic differences that are not directly attributable to local sequence changes. As previously reported [50] there are several large seemingly non-polymorphic regions in B73 relative to Mo17. These are likely identical-by-descent (IBD) regions that represent shared inheritance of the same haplotype block in these two different inbred genotypes from a common parent [50]. We analyzed 10 putative IBD regions in the B73-Mo17 genome that are at least 2 Mb in length, have no evidence for structural variation, and have extremely low SNP densities (Table 4). The SNP rates in these regions (1 every 44.2 kb) are below the levels of sequence error rates reported for the B73 reference genome [14]. Despite the near-absence of genetic variation within these regions there are 52 differentially methylated probes and 9 DMRs within these regions. The large low diversity region on chromosome 8 provides several examples of altered methylation levels within a large region that lacks sequence differences (Figure 3B and 3C). We used the Mo17 whole-genome shotgun sequences and targeted PCR to confirm that absence of any InDels within 2 kb of each of the nine DMRs located within the IBD regions. The majority (8/9) DMRs in IBD regions did not have any InDels. Only one of these DMRs in an IBD region exhibit sequence polymorphism. At this DMR there is evidence for a recent insertion of a repetitive element in the B73 allele and B73 is more highly methylated than Mo17.

**Table 4:** IBD regions in the B73-Mo17 genome.

Chromosome	Start (Mb)	Stop (Mb)	Length (Mb)	Fold reduction in SNP diversity	Variable methylation probes	Variable methylation segments
1	116	119	3	49.1	0	0
2	86	88	2	31.4	1	1
2	136.5	140.5	4	30.2	2	0
2	178.5	185	6.5	42.2	5	0
3	162.5	165	2.5	40.9	4	2
4	126	130	4	39.3	1	0
4	163	166.5	3.5	25.0	1	1
5	54	56	2	25.4	2	1
5	206	210	4	27.3	11	0
8	142.5	160	17.5	36.3	25	4

### Characterization of inheritance for differential methylation

A set of 33 DMR regions consisting of 14 regions of B73 hypermethylation and 19 regions of Mo17 hypermethylation was selected for further characterization (Table 5). These included eight DMRs that were present in the IBD regions. Quantitative PCR-based assays were developed to assess relative methylation levels following digestion with the methylation dependent enzymes MspJI and FspEI. The methylation differences observed in the full-genome profiling were confirmed for 28/33 of these regions in independent biological samples of B73 and Mo17 DNA (Table 5). The differential methylation was also assessed using the methylation-sensitive enzymes HpaII and/or PstI for ten of these same DMRs (Table S5) including the three that had not been supported by methylation-dependent digests. All DMRs (4/4) that include a PstI (CHG sensitive) site were validated and 8 of the 10 DMRs that had a HpaII site were validated (Table S5). Two of the three regions that were not conclusively validated by the methylation-dependent enzyme digests did exhibit differential methylation when tested with HpaII. The other DMR was not supported by assays with either methylation-dependent or –sensitive enzymes. The classification of differential methylation was also supported by an analysis of read counts from methyl-sensitive and insensitive sequencing libraries from Gore et al [47].

**Table 5:** Characterization of variable methylation segments.

segID	Chr	Start	Stop	Assay	Mspj1 relative methylation^a^	FspEI relative methylation^a^	Confirmed?	IBD region?	Cis/trans control
4	chr1	86899	87699	MDMR_40	−9.23	−9.14	Yes		cis
36	chr1	19,520,608	19,522,008	MDMR_43	−2.84	−7.66	Yes		
42	chr1	19982857	19984474	MDMR_82	−3.79	−5.22	Yes		
94	chr1	39036362	39039562	MDMR_24	−5.14	−5.87	Yes		trans
213	chr1	160563568	160563968	BDMR_22	2.73	2.44	Yes		cis
215	chr1	162,538,673	162,540,073	MDMR_44	−7.81	−11.09	Yes		
247	chr1	190761322	190762322	MDMR_27	−5.65	−10.1	Yes		trans
710	chr2	144,048,581	144,048,981	BDMR_45	−1.38	−0.72	No		
788	chr3	7,683,400	7,686,600	MDMR_73	−3.7	−8.04	Yes	Yes	cis
792	chr3	8,346,040	8,348,062	MDMR_74	−6.36	−9.79	Yes	Yes	cis
794	chr3	8,357,591	8,358,191	BDMR_78	3.76	9.4	Yes	Yes	
999	chr3	183,380,573	183,381,173	BDMR_3	−0.82	−0.17	No		
1060	chr3	205,814,185	205,816,207	MDMR_41	−6.3	−13.2	Yes		
1064	chr3	206,677,794	206,678,782	BDMR_48	1.9	3.34	Yes		
1066	chr3	209,258,739	209,259,431	BDMR_51	1.31	3	Yes		
1193	chr4	108,425,289	108,425,889	BDMR_79	−6.21	1.17	No	Yes	
1252	chr4	140,005,016	140,005,441	MDMR_1	−4.73	−3.93	Yes		cis
1280	chr4	160,954,336	160,955,336	MDMR_37	−5.36	−9.87	Yes		cis
1479	chr5	69,250,995	69,252,274	BDMR_49	5.58	4.69	Yes		cis
1493	chr5	96,853,806	96,854,606	BDMR_53	3	1.64	Yes	Yes	cis
1603	chr6	60,183,875	60,184,275	MDMR_8	−4.9	−3.95	Yes		
1697	chr6	161,113,703	161,114,709	MDMR_4	−4.34	−9.87	Yes		
1840	chr7	150,216,544	150,218,384	BDMR_31	2.94	2.02	Yes		
1895	chr8	97,274,412	97,276,412	MDMR_36	−3.58	−1.43	No		
1938	chr8	143,704,696	143,705,542	MDMR_75	−5.34	−10.08	Yes	Yes	
1940	chr8	145,875,662	145,877,862	MDMR_76	−3.41	−2.52	Yes	Yes	cis
1946	chr8	151,080,956	151,083,158	MDMR_77	−1.34	ND	Yes	Yes	
1968	chr9	3,855,741	3,856,077	BDMR_62	2.84	2.9	Yes		
2005	chr9	20,864,861	20,865,197	MDMR_13	−2.25	−1.99	Yes		
2033	chr9	37,257,975	37,258,311	BDMR_59	2.52	−0.83	No		
2178	chr9	116,238,414	116,238,750	BDMR_32	2.9	2.93	Yes		
2209	chr9	145,760,392	145,760,896	BDMR_47	3.06	3.89	Yes		cis
526	chr10	126586236	126586636	MDMR_38	−5.66	−6.09	Yes		trans

a The relative methylation is calculated as the (B73 mock Ct - B73 digest Ct) - (Mo17 mock Ct - Mo17 digest Ct). Values above zero reflect higher methylation in Mo17 while values below zero reflect higher methylation levels in B73.

b The number of methylated and unmethylated inbreds (from Figure 4B) is reported.

We assessed relative methylation levels for 13 of the DMRs in selected genotypes from a population of near-isogenic lines (NILs) derived from B73 and Mo17 [58]. The levels of methylation in NILs can be used to evaluate the relative contribution of linked and unlinked genomic regions and to test for paramutation-like transfer of information between alleles. The expected results for each of these potential scenarios are shown in Figure 4A. The genotype for the chromosomal region containing the DMR is expected to predict the methylation state in the NIL if the methylation change is purely epigenetic or if linked sequence polymorphisms regulate methylation levels. Alternatively, if unlinked genomic regions are directing the methylation levels at DMRs introgressed into a NIL then the DMR is expected to exhibit methylation levels similar to the recurrent parent. For each of the DMRs we selected several genotypes that provided an introgression of the locus into either a B73 or Mo17 genomic background. In addition, as a control we monitored DNA methylation levels in several NIL genotypes that did not have an introgression at the locus of the DMR. In general, the control assays show a high stability of DNA methylation levels at these DMRs. The analysis of the NILs with introgressions at the DMR loci reveal that 10/13 have methylation levels that can be predicted by the haplotype of the region surrounding the DMR. This could reflect stable inheritance of epigenetic variation or *cis*-linked genetic changes that are directing the methylation difference. The other three DMRs that were mapped have DNA methylation patterns that are influenced by genomic regions that are unlinked to the DMR locus, suggesting that the methylation levels of these loci are controlled by *trans*-acting loci. Four (of the 13) DMRs that were mapped are located within the IBD regions and each of these exhibited methylation patterns that were controlled by *cis*-linked regions despite the absence of closely linked genetic variation within these regions.

**Figure 4:**
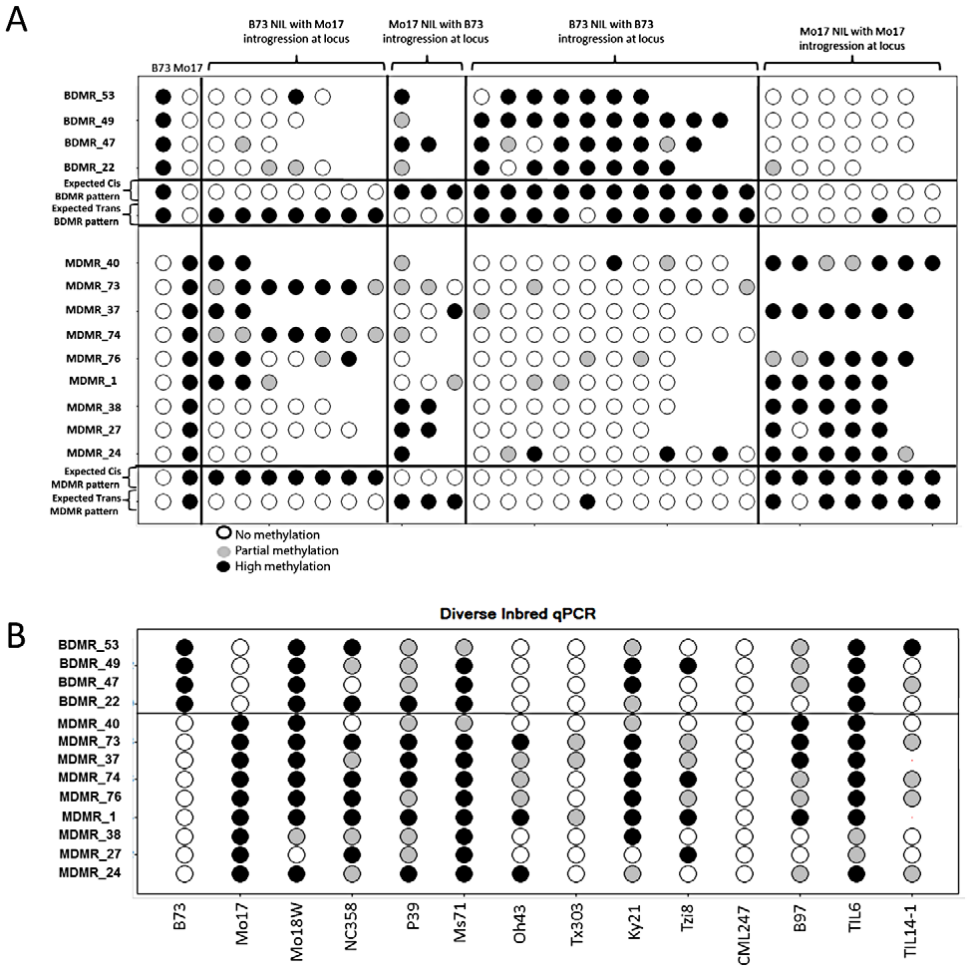
Variable DNA methylation patterns in near-isogenic lines and diverse inbreds. (A) The relative DNA methylation levels in selected near-isogenic lines was tested by digestion with the methylation dependent restriction enzyme MspjI followed by qPCR. Different subsets of NILs were selected and analyzed for each of 13 DMRs. The first two columns show the data from B73 and Mo17. Open circles reflect low methylation levels and black circles indicate high methylation levels. Intermediate methylation levels are indicated by gray color. The next group of 2–7 genotypes show the data from NILs that have B73 as the recurrent parent (>95% of the genome) and have introgression of the Mo17 haplotype in the region containing the DMR. The variable number of genotypes tested reflects the fact that some DMR loci are have more NILs with an introgression than others. The next group of 1–3 genotypes are NILs that are primarily Mo17 but have B73 introgressed at the DMR. The next two groups provide “control” genotypes of B73-like or Mo17-like NILs that do not have an introgression at the DMR. The expected patterns for *cis* (local) inheritance of DNA methylation or *trans* (unlinked) control of DNA methylation are shown. Note that the expected pattern for *trans* control would include a small number of genotypes with the methylation pattern from the introgressed genotype in cases where the trans-acting locus is introgressed. (B) The same type of assays were performed on a panel of 12 diverse inbred genotypes, including two inbred teosinte lines, to monitor the frequency for the hyper- and hypo-methylated states.

The relative DNA methylation patterns for these 13 DMRs were also assessed in a panel of 10 other inbred lines of maize and two teosinte inbred lines (TILs) (Figure 4B). Each of these DMRs exhibits at least one other genotype with high or low levels of methylation indicating that the B73-Mo17 states are not unique within maize.

## Discussion

Maize has a rich history of serving as a model for epigenetic studies. The first examples of imprinting and paramutation were discovered in maize [59], [60] and there have been a number of pioneering studies on the epigenetic regulation of transposable element in maize [61], [62]. While these discoveries have been enabled by the ease of genetic studies in maize it is also likely that the complex organization of the maize genome with many interspersed transposons and genes has led to numerous examples of epigenetic regulation. In this study we have performed a genome-wide characterization of DNA methylation levels in two inbred lines of maize and found hundreds of loci with differences in DNA methylation levels. This study of natural epigenetic variation also demonstrates the utility of near-isogenic lines for characterizing epialleles.

### Methylation dynamics along maize chromosomes and genes

Our data provide evidence for higher levels of DNA methylation in the pericentromeric regions of maize chromosomes. This is quite similar to observations in Arabidopsis [18]–[20]. Although there is a general negative correlation between methylation density and recombination, methylation density does not exactly mirror recombination rates. Recombination happens much more frequently near the ends of the chromosomes and quickly drops to a lower level internally [54]. The DNA methylation patterns exhibit a much more gradual change along the length of the chromosomes, potentially suggesting that the differences in recombination rate along the length of a chromosome are not directly related to DNA methylation levels. In general, the methylation patterns within maize gene bodies are similar to the density of CG sites. However, it is clear that the methylation levels in regions immediately upstream and downstream of gene bodies are not reflective of CG density. Interestingly, while short maize genes have elevated CG content throughout the gene body the longer maize genes have elevated CG content only in the first 500–1000 bp.

We also noted very distinct patterns of DNA methylation in maize genes. In general, methylation is lower in high-confidence genes (i.e., members of the FGS), highly expressed genes and genes in syntenic positions relative to other grass species. Putative genes that were rejected during stringent genome annotation (often partial length sequences or putative transposons) are more highly methylated. The lack of detectable methylation differences between genes located in the maize1 and maize2 sub-genomes was surprising because there is evidence that maize1 genes are generally more highly expressed than maize2 genes [57]. Therefore, DNA methylation is unlikely to provide the mechanism for controlling the expression differences between the two sub-genomes of maize. The significant difference in average methylation levels for syntenic and non-syntenic genes is correlated with lower expression levels for non-syntenic genes. The non-syntenic genes that are conserved among the grasses but exhibit different genomic positions are the result of gene movement, perhaps mediated by transposons. Approximately 1/3 of the maize FGS genes are not in syntenic positions relative to other grasses, yet nearly all genes investigated by classical genetics to date belong to the fraction of the genome located in syntenic positions [63]. Their higher levels of methylation may reflect the presence of transposon sequences near these genes or may result from the insertion of a gene into new chromosomal environments that are lacking some of their ancestral regulatory sequences.

### Many stable DMRs are found in B73 and Mo17

Several groups have demonstrated that perturbation of epigenetic information can affect quantitative traits [28]–[31]. In addition, the existence of natural epigenetic variation has been demonstrated for individual loci [32]–[44] or chromosomes [8]. A primary objective of this study was to document the prevalence and distribution of variable methylation levels in different maize genotypes. There are hundreds of examples of differential methylation in B73 and Mo17. In general, many of these DMRs are located in intergenic regions and may reflect differences in transposon silencing among the genotypes. However, at least 71 of the 690 variable methylation regions are found within 500 bp of a high-confidence gene (FGS). Following the discovery of these regions we were able to pursue further characterization using a population of NILs. The NILs provide a useful tool for assessing the stability of DNA methylation patterns and for testing whether the epigenetic variation is caused by genetic differences elsewhere in the genome.

The analysis of DNA methylation levels at several DMRs within the NILs addressed the stability of the DNA methylation patterns. The near-isogenic lines were developed by three back-crosses followed by at least four rounds of self-pollination [59]. In an analysis of the control lines (lines without an introgression at the DMR locus for eleven *cis*-controlled DMRs) there is evidence for stable inheritance as 85% of the assays reveal the expected methylation level. There are a small number of assays (5/150) that exhibit a completely changed methylation state and another 17/150 exhibit a partial gain or loss of DNA methylation. These examples may reflect inaccuracies in our measurements of DNA methylation or actual instability of DNA methylation patterns. In general, we observe relatively stable inheritance with rare examples of both gains and losses of DNA methylation. We did not observe evidence for paramutation-like effects where methylation levels were affected by heterozygosity for the DMRs.

Stable differences in DNA methylation levels between two genotypes can be the result of differences in epigenetic state that are faithfully propagated to offspring. Alternatively, they may be the result of genetic changes elsewhere in the genome that direct epigenetic modifications at an unlinked site. For example, structural rearrangements of the PAI1-PAI4 locus on chromosome 1 of Arabidopsis control the methylation state of the two other PAI loci on chromosomes 1 and 5 [36]. The conditioning of DNA methylation state by either linked or unlinked genomic regions would be examples of obligatory epialleles where a genetic change at one locus programs variable methylation at another locus. We studied the contribution of linked and unlinked genomic regions to the methylation differences between B73 and Mo17 for 13 of the DMRs using a series of near-isogenic lines [58]. Three of the regions exhibit evidence for *trans*-acting control of DNA methylation patterns. The remaining ten loci have methylation patterns that are either stably inherited or are continuously directly by local sequence changes.

### Evidence for pure epialleles in maize

A major unresolved question about epigenetic variation is whether the majority of epigenetic variation exhibits strong linkage disequilibrium with nearby genetic differences [9]. If genetic markers, such as SNPs, are in strong linkage disequilibrium with epigenetic changes then the functional consequences of epigenetic differences would likely be revealed by assays of linked genetic differences. In particular, “obligatory” epialleles are entirely conditioned by nearby genetic changes [5]. Alternatively, “facilitated” epialleles exhibit stochastic variation in epigenetic state with a conditioning genetic change, and “pure” epialleles exhibit stochastic variation in epigenetic state independent of any genetic changes [5]. Both facilitated and pure epialleles will show differences in epigenetic state that are not completely linked to, or predicted, by nearby genetic polymorphisms.

We were interested in whether some of the epigenetic changes between B73 and Mo17 might be due to epigenetic changes that are not directly caused by nearby genetic differences such as transposon insertions or by unlinked rearrangements that might direct methylation in *trans* via RNA-directed DNA methylation. This led us to focus on the 10 extended B73-Mo17 identical-by-descent regions. These regions are most likely the result of shared inheritance of a chromosomal region from a genotype that was used in the pedigree of both B73 and Mo17. At least one genotype, CI187-2, is present in the pedigree of both B73 and Mo17 [64]. It is therefore possible that these regions could exhibit identity by descent (IBD). It is worth noting that other maize genotypes have alternative haplotypes in these regions so they are not the result of large selective sweeps among all maize genotypes [65]. The absence of detected structural variation and few SNPs within these regions suggest that any observed epigenetic differences between B73 and Mo17 are not the result of nearby genetic polymorphisms. We found 9 DMR (of 690 genome-wide) within these IBD regions and only one of these has evidence for a nearby genetic change. The number of DMRs within the IBD regions (9) is very close to the number we would have expected (13) based on the frequency per Mb within the whole genome. The finding that all four of these regions that were assessed in NILs show stable inheritance provides evidence for heritable epigenetic information in the absence of genetic differences. Our initial focus on IBD regions allowed the discovery of epigenetic variation without nearby genetic changes given the extended regions of identity. However, it is likely that many of the DMRs that are located in non-identical by descent genomic regions may also be the result of purely epigenetic changes. We noted that one-third of the 690 DMRs do not contain any SNPs in B73 relative to Mo17 within 1 kb of the DMR and may represent epigenetic differences that are not conditioned by genetic differences.

This study provides a detailed view of the distribution of cytosine methylation in two maize inbreds. The evidence for faithfully inherited methylation differences, even in the absence of nearby genetic polymorphisms, provide evidence for at least partially stable epigenetic variation in maize that would not be revealed by high-resolution analyses of genetic differences. There are likely functional consequences of the altered methylation levels in B73 and Mo17. There are several examples in which a DMR within an identical by descent region is near the promoter for a FGS gene and several of these genes exhibit differential expression in other tissues of B73 and Mo17 (data not shown). Further characterization of the relationship between expression variation and methylation variation may identify examples of epigenetic variation that affect phenotypic differences among inbred lines. This study, in combination with recent analyses of epiRILs in Arabidopsis [28]–[31], provides evidence for heritable epigenetic information that may contribute to quantitative trait differences within species. Future research is required to uncover evidence for the contribution of the variable methylation we have described in this study to phenotypic differences among maize genotypes.

## Materials and Methods

### Plant materials and DNA isolation

Three replications of B73 and Mo17 seedlings were grown in a randomized block design. The seeds for each replication came from a unique, single source (ear). For each replication, 10 seedlings were grown in pots (5 seedlings per pot) that were assigned random positions. Seedlings were grown under controlled conditions in a greenhouse at the University of Minnesota (St. Paul, MN) with a light cycle of 15 hours lights on and 9 hours lights off each day. Seedlings were watered daily as needed. After 18 days of growth, the 3rd leaf (L3) of each plant was harvested and pooled with other plants from the same pot/replication and immediately frozen in liquid nitrogen. DNAs were isolated using the CTAB method. Phenol∶chloroform extraction and subsequent precipitation in 0.1× volume Na-Acetate (3 M) and 2× volume 100% EtOH was conducted to purify the DNA samples. 15–30 ug of gDNA in 650–700 uL nuclease-free water was sonicated for five, ten- second pulses as per the methods of [66]. Samples were quantified and run on 1.5% agarose gels to verify that DNAs were fragmented to 200–400 bp.

### Array design and annotation

A NimbleGen 2.1 M feature long oligonucleotide array was designed using B73 RefGen2 assembly (provided by the Arizona Genomics Institute). The maize genome exhibits a complex architecture with many repetitive sequences interspersed with low-copy genic sequences [14]. A repeat masked version of the pseudomolecule sequences from RefGenv2 of the B73 genome (provided by J Stein and D Ware) were used to design probes to low-copy regions. Thermally balanced probes were designed every ∼200 bp across the low-copy portion of the maize genome. The actual spacing varies in some cases to allow for ideal probe selection. In addition, a higher density of probes (one probe every ∼56 bp) was used for chromosome 9 to determine whether higher probe density provided increased resolution for methylation detection (Table S1). The probes were each annotated with respect to their copy number in the B73 genome, the number of close matches and their location relative to gene models. Syntenic orthologs of maize genes in sorghum, rice, and brachypodium were identified using the combined synonymous substitution rate of syntenic blocks method described in [57]. A maize gene was considered to be recently inserted if orthologous locations could be identified in rice, sorghum, and brachypodium by the syntenic conservation of up and downstream genes, but no homologous gene nor unannotated homologous sequence was identified in any species at the predicted orthologous location.

### Immunoprecipitation of methylated DNA, labeling, and hybridization

Methylated DNA was immunoprecipitated with an anti-5-methylcytosine monoclonal antibody from 400 ng sonicated DNA using the Methylated DNA IP Kit (Zymo Research, Orange, CA; Cat # D5101). For each replication and genotype, whole genome amplification was conducted on 50–100 ng IP DNA and also 50–100 ng of sonicated DNA (input control) using the Whole Genome Amplification kit (Sigma Aldrich, St. Louis, MO, Cat # WGA2-50RXN). For each amplified IP input sample, 3 ug amplified DNA were labeled using the Dual-Color Labeling Kit (Roche NimbleGen, Cat # 05223547001) according to the array manufacturer's protocol (Roche NimbleGen Methylation UserGuide v7.0). Each IP sample was labeled with Cy5 and each input/control sonicated DNA was labeled with Cy3. The no anti-body control (negative control) was also labeled, using the average volumes required for the experimental samples. Samples were hybridized to the custom 2.1 M probe array (GEO Platform GPL13499) for 16–20 hrs at 42°C. Slides were washed and scanned according to NimbleGen's protocol for the GenePix4000B scanner. Images were aligned and quantified using NimbleScan software (Roche NimbleGen) producing raw data reports for each probe on the array.

### Normalization and linear modeling

Pair files exported from NimbleScan were imported into the Bioconductor statistical environment (http://bioconductor.org/). Microarray data channels were assigned the following factors: B73, Mo17, B73 input, or Mo17 input depending on sample derivation. Non-maize probes and vendor-supplied process control probes were configured to have analytical weights of zero. Variance-stabilizing normalization was used to account for array-specific effects. Factor-specific hybridization coefficients were estimated by fitting fixed linear model accounting for dye and sample effects to the data using the limma package [67]. To compute biologically relevant information about B73 and Mo17 DNA methylation, the following contrasts were then computed: B73 IP vs B73 input (B73 methylation); Mo17 input vs B73 input (CGH and differential hybridization efficiency); Mo17 IP vs [Mo17 input vs B73 input] (Mo17 methylation corrected for differential hybridization efficiency); B73 IP vs [Mo17 IP vs [Mo17 input vs B73 input]] (differential DNA methylation corrected for differential hybridization efficiency). Moderated t-statistics and the log-odds score for differential MeDIP enrichment were computed by empirical Bayes shrinkage of the standard errors with the False Discovery Rate controlled to 0.05 [67]. Full results are available for download from the following URL: http://genomics.tacc.utexas.edu/data/eichten-plos-genetics-2011-a. Microarray results were deposited with NCBI GEO under accession GSE29099.

### Defining CGH copy-number variations

CGH data were obtained from the hybridization of un-enriched B73 and Mo17 genomic DNA (B73 and Mo17 input channels). DNAcopy [52] was performed to identify segments showing similar hybridization patterns based on chromosomal order of probes. Defined segments were analyzed by expectation maximization model analysis [52], [68] to identify segments that fall into three predicted sub-distributions with non-uniform variances. For each segment, the posterior probability that it occurred in each of the three distributions was determined. Segments that had >0.95 probability of falling into either the first or third sub-distributions were defined as containing more copies in Mo17 than in B73 (M>B CNV) or significant fewer to no copies in Mo17 relative to B73 (M<B CNV and PAV) respectively.

### Analysis of variable methylation

To define probes with differential methylation between the B73 and Mo17 inbreds, the significance values developed from the B73 vs Mo17 relative methylation linear model probes were used. Probes with a significance value of <0.001 were considered differentially methylated between the two inbreds. The direction of the variation was determined based on the positive or negative value of the B73 methylation minus the Mo17 methylation state. A total of 4172 B73 hypermethylated and 5367 Mo17 hypermethylated probes were classified using this method.

To identify segments showing differential methylation between B73 and Mo17, the DNAcopy algorithm [52] was used on 1,088,820 Mo17 unique probes in the B73 vs. Mo17 relative methylation linear model results. The EM algorithm [53], [68] was used to estimate the mixing proportion, mean, and variance associated with three predicted sub-distributions with non-uniform variances found within the B73 vs Mo17 segments. For each segment, the posterior probability that it occurred in each of the three distributions was determined. Segments that had >0.95 probability of falling into either the first or third sub-distributions were called as Mo17 hypermethylated or B73 hypermethylated segments respectively.

### qPCR

Quantitative PCR (qPCR) was performed to evaluate the efficiency of 5-methylcytosine immunoprecipitation using regions of the *Mez1* gene known to have methylation (5′region) and non-methylation (Exon 9) [66]. Primers were designed within the methylated region (forward primer 5′- TTGTGTCGAGGTCTCGAATG-3′, reverse primer 5′- TGTTGAAGCGCATTAGCACT -3′) and within the non-methylated region (forward primer 5′- CAACAAAGTGAAAGCTCTTCAACTGCAA-3′, reverse primer 5′-CACAACACTCCCCTAGTCCCTCAAAAGTT-3′). Primer amplification and efficiency were tested in B73 and Mo17 genomic DNA. Three technical replications were included for each of two biological replications of B73 and Mo17 IP and input DNA samples. The relative amount of immunoprecipitated DNA (percentage of the input control DNA for each sample) was calculated (Figure S1). As expected, the IP negative control and qPCR no template controls either did not amplify or amplified approximately 10 cycles after the experimental samples (>1000 fold difference, data not shown). *Mez1* qPCR reactions were conducted using 100 ng DNA and Light Cycler480 SYBR Green I Master (Roche, Cat # 04707516001) on the LightCycler480 instrument (Roche) in accordance with Roche's protocol for SYBR Green on the LightCycler480.

Primers were designed for 33 regions within DMRs (Table S6). 1 microgram of genomic DNA was digested for 16 hr with MspJI or FspEI (New England Biolabs). Mock digestions were performed substituting glycerol for restriction enzyme. qPCR reactions were performed using 37 ng DNA and SsoFast EvaGreen Supermix (BioRad) on the Chromo4 instrument (BioRad) in accordance with SsoFast protocol. The difference between digest C(t) and mock C(t) was calculated for each genotype tested. As our selected enzymes target methylated cytosines, higher methylation leads to increased digestion and subsequently longer C(t) times. DMRs in B73 and Mo17 were validated as higher methylation levels for larger differential C(t) value between the inbred lines. NIL and diverse inbred samples were compared across individual primer pairs and methylation state was determined by comparing C(t) difference values.

## Supporting Information

Figure S1 Enrichment of methylated DNA by immunoprecipitation. (A) The percent of input DNA recovered following 5-methylcytosine immunoprecipitation of three biological replicates of B73 was determined for two different regions by qPCR. The unmethylated region is 5,270 to 5,380 of Mez1 (exon 9) and the methylated region is from −1,238 to −1,038 of Mez1 [48]. Very similar enrichments were observed for Mo17 (Haun et al., 2007). (B) A density plot is used to visualize the distribution of all B73 log2(IP/input) values (black dotted line). This observed distribution can be approximated by an expectation maximization model that assumes three normal distributions (solid lines that add up to the red dashed line). Values with a high posterior probability of being sampled from the black distribution are assigned as methylated.
(TIF)

Figure S2 Examples of UpCNV and PAV probes showing both high and low levels of DNA methylation in B73 (A) and Mo17 (B). Regions of decreased and increased methylation levels for PAV (B>M, Blue) and UpCNV (M>B, Red) loci are present throughout chromosome 8. Variable methylation of PAV and UpCNV also occur throughout the chromosome (C).
(TIF)

Figure S3 Copy number and genomic structural variation effects on methylation levels. (A) The distribution of B73 methylation values is shown for all probes as well as for probes in B>M segments and a subset of B>M segments that likely represent PAV sequences as the Mo17 signal is substantially lower than the B73 signal. There are no significant differences in the average methylation levels of these probes. In (B) and (C) the methylation of M>B probes is shown for B73 and Mo17, respectively. These likely represent sequences with copy number gains in Mo17 relative to B73 but there is not a substantial differences in the methylation of these sequences relative to other genomic sequences. (D) A boxplot is used to show the distribution of B73 methylation values for all probes with 1, 2, 3, or 4+ copies in the B73 genome. The methylation level significantly increases as the number of perfect matches increases. (E) A similar plot is used to show how the number of close (>90% identity and coverage) matches is similarly related to increased methylation values.
(TIF)

Figure S4 Percent methylation across maize chromosomes. The percentage of methylation is plotted as a 5 Mb window sliding 1 Mb downstream across each of the 10 maize chromosomes. Blue and red lines indicate B73 and Mo17 percent methylation respectively. The green line indicates the centromere position of each chromosome. All other tracks are the same as in Figure 1C.
(TIF)

Figure S5 Increased methylation at rejected genes. The genes in the working set that were rejected from the FGS include possible contamination (bacterial sequences), low confidence FGENESH models, probable transposons and probable pseudogenes. Genes in each of these categories exhibit significantly higher methylation levels than genes in the FGS.
(TIF)

Figure S6 High levels of methylation within gene body. (A) The FGS genes were divided into different length categories to assess the level distribution of gene body methylation. (B) length categories also show increased CpG dinucleotide sites within the gene body. (C) Methylation levels and CpG dinucleotide proportions show related patterns within gene bodies. Methylation and CpG proportion diverge when not within genic sequence. (D) Methylation levels are higher in intergenic sequences than in exons and introns. The lowest levels of methylation are observed in introns and at exon/intron boundaries. (E) A profile of the methylation patterns along genes for only exon (black) or intron (red) shows that gene body methylation at the 5′ end of genes is confined to exons. Similarly, the reduced methylation at the 3′ end of genes is more pronounced in exons than in introns.
(TIF)

Figure S7 (A) Boxplot showing the different methylation levels between expression quartiles. Total number of probes in each category from the B73_unique probe set are presented under each category. Tukey HSD results are provided in gray box. (B) Methylation levels are not affected by sub-genome 1 and 2. The FGS genes were all classified based on whether they were located in regions of the maize genome classified as sub-genome 1 or sub-genome 2 (Based on [57]). There is no evidence for altered methylation levels for genes in sub-genome 1 relative to sub-genome 2.
(TIF)

Figure S8 Characterization of maize DMRs. (A) A histogram is used to show the distribution of the length of the DMRs identified in B73 relative to Mo17. (B–D) The DMRs were analyzed to assess enrichments for syntenic positioning (B), subgenome classification (C), and expression quartile (D). For each comparison, the proportion of differentially methylated genes in each selected category were contrasted against the total number of genes in the filtered gene set.
(TIF)

Table S1 Number of probes on each chromosome.
(XLS)

Table S2 Identification of PAV and CNV in Mo17 relative to B73.
(XLS)

Table S3 Number of similar and identical matches per probe.
(XLS)

Table S4 List of all DMRs identified.
(XLS)

Table S5 Summary of validations of DMRs by restriction-sensitive restriction digests.
(XLS)

Table S6 Primers used for qPCR validations.
(XLS)
