# Supplementary figures and images for: Heritable Epigenetic Variation among Maize Inbreds
Source: PLoS Genet. 2011 Nov 17;7(11):e1002372. doi: 10.1371/journal.pgen.1002372 (PMC3219600; doi:10.1371/journal.pgen.1002372)

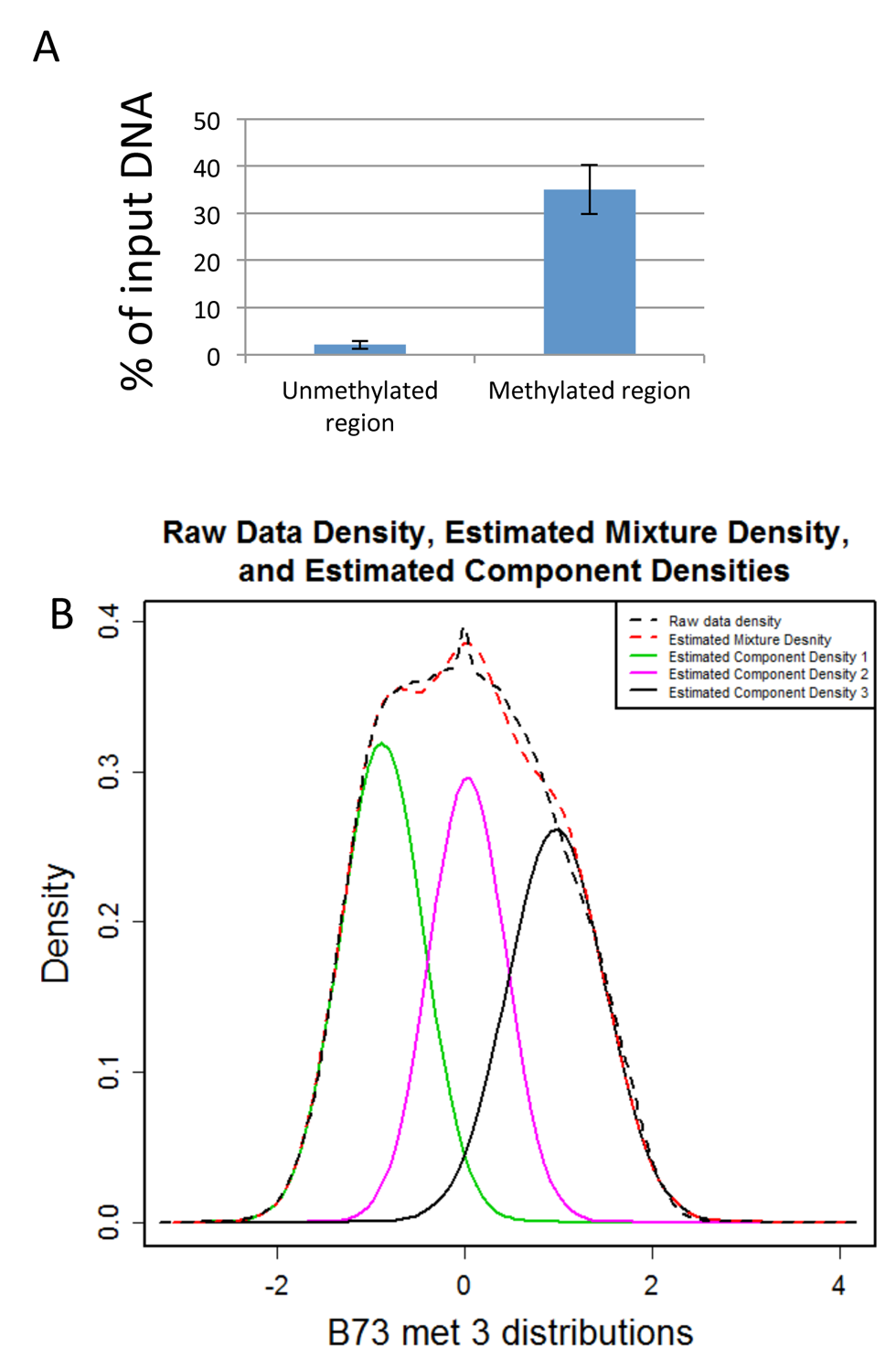

Supplement: Figure S1 — Enrichment of methylated DNA by immunoprecipitation. (A) The percent of input DNA recovered following 5-methylcytosine immunoprecipitation of three biological replicates of B73 was determined for two different regions by qPCR. The unmethylated region is 5,270 to 5,380 of Mez1 (exon 9) and the methylated region is from −1,238 to −1,038 of Mez1 [48]. Very similar enrichments were observed for Mo17 (Haun et al., 2007). (B) A density plot is used to visualize the distribution of all B73 log2(IP/input) values (black dotted line). This observed distribution can be approximated by an expectation maximization model that assumes three normal distributions (solid lines that add up to the red dashed line). Values with a high posterior probability of being sampled from the black distribution are assigned as methylated. (TIF) [file pgen.1002372.s001.tif]

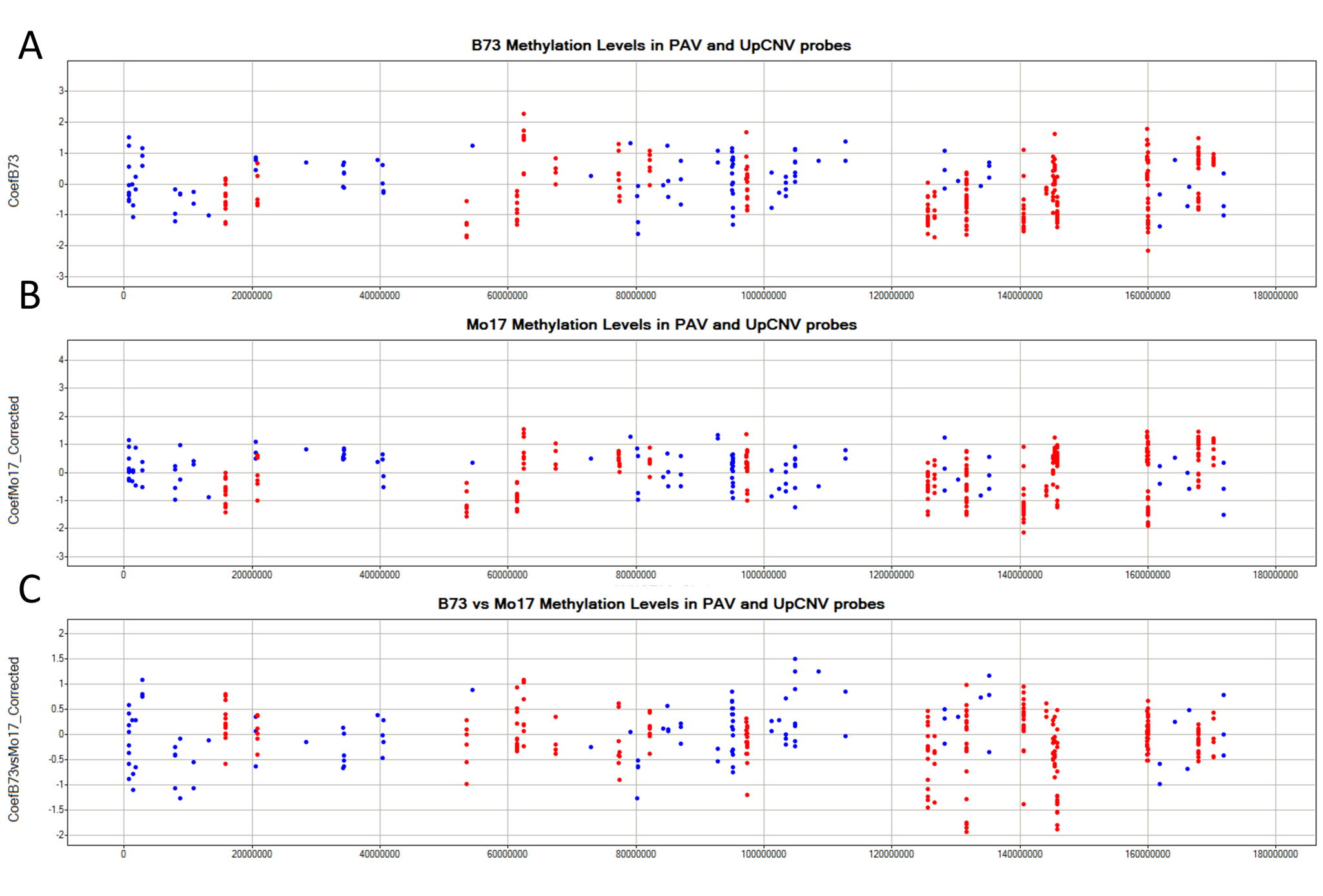

Supplement: Figure S2 — Examples of UpCNV and PAV probes showing both high and low levels of DNA methylation in B73 (A) and Mo17 (B). Regions of decreased and increased methylation levels for PAV (B>M, Blue) and UpCNV (M>B, Red) loci are present throughout chromosome 8. Variable methylation of PAV and UpCNV also occur throughout the chromosome (C). (TIF) [file pgen.1002372.s002.tif]

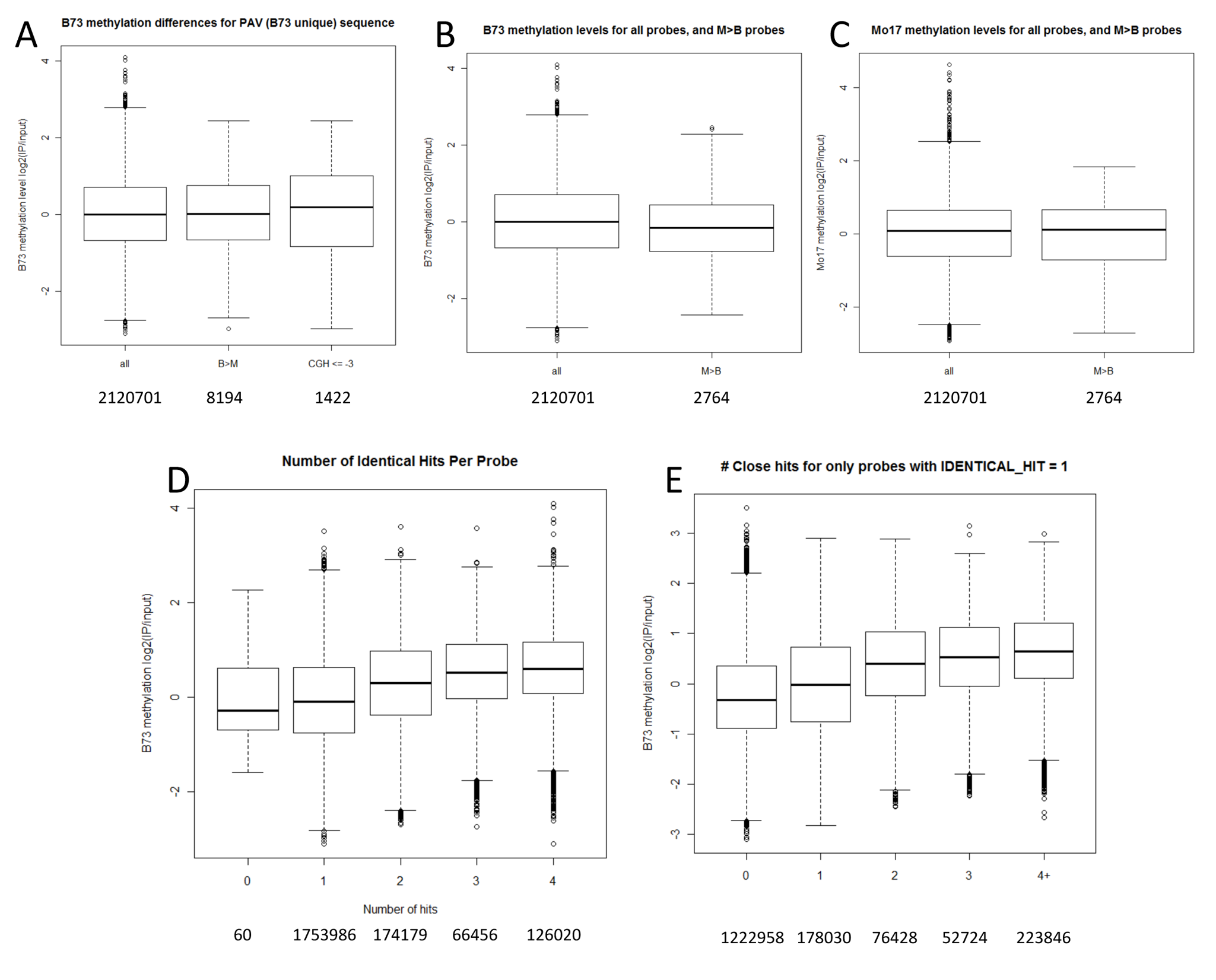

Supplement: Figure S3 — Copy number and genomic structural variation effects on methylation levels. (A) The distribution of B73 methylation values is shown for all probes as well as for probes in B>M segments and a subset of B>M segments that likely represent PAV sequences as the Mo17 signal is substantially lower than the B73 signal. There are no significant differences in the average methylation levels of these probes. In (B) and (C) the methylation of M>B probes is shown for B73 and Mo17, respectively. These likely represent sequences with copy number gains in Mo17 relative to B73 but there is not a substantial differences in the methylation of these sequences relative to other genomic sequences. (D) A boxplot is used to show the distribution of B73 methylation values for all probes with 1, 2, 3, or 4+ copies in the B73 genome. The methylation level significantly increases as the number of perfect matches increases. (E) A similar plot is used to show how the number of close (>90% identity and coverage) matches is similarly related to increased methylation values. (TIF) [file pgen.1002372.s003.tif]

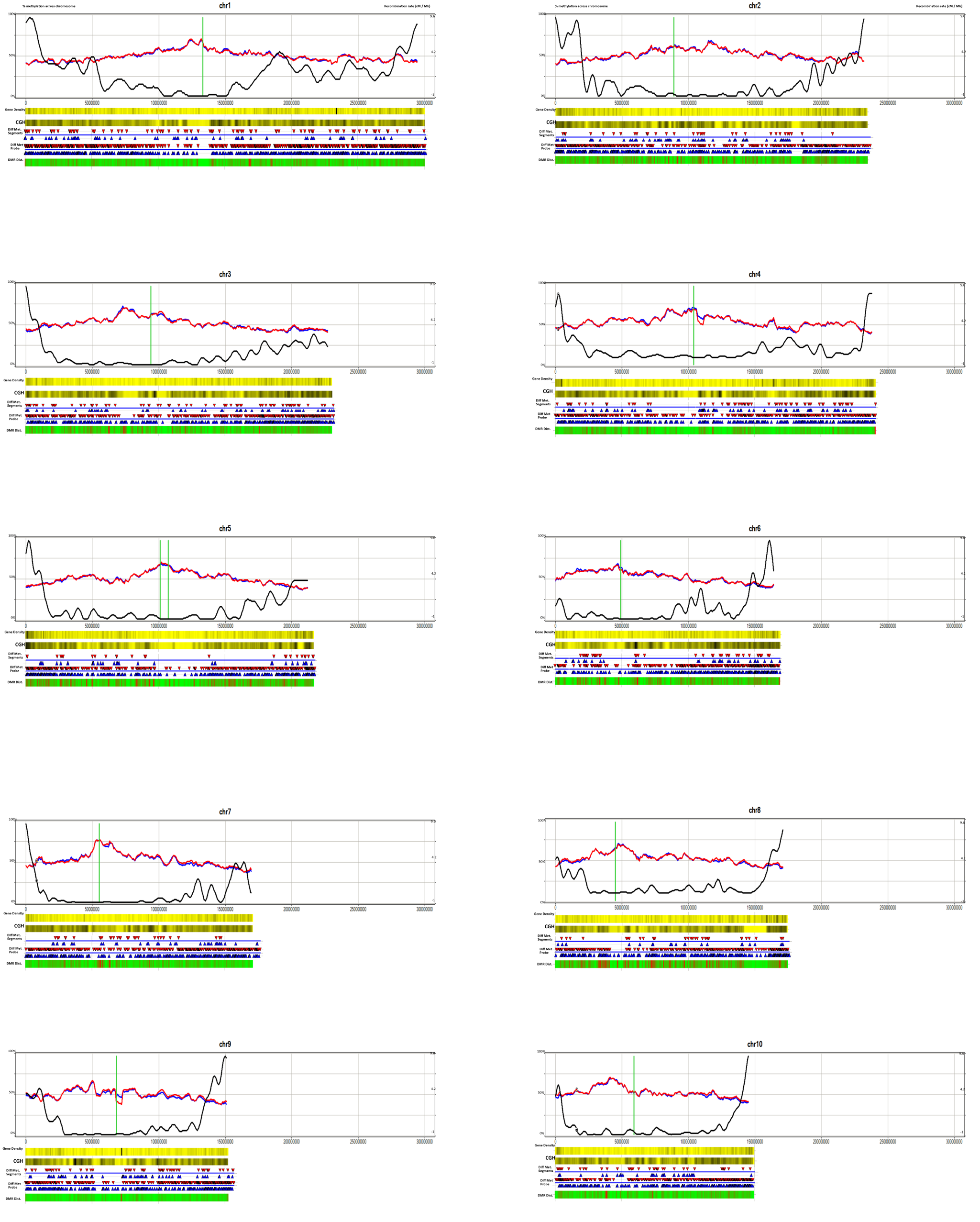

Supplement: Figure S4 — Percent methylation across maize chromosomes. The percentage of methylation is plotted as a 5 Mb window sliding 1 Mb downstream across each of the 10 maize chromosomes. Blue and red lines indicate B73 and Mo17 percent methylation respectively. The green line indicates the centromere position of each chromosome. All other tracks are the same as in Figure 1C. (TIF) [file pgen.1002372.s004.tif]

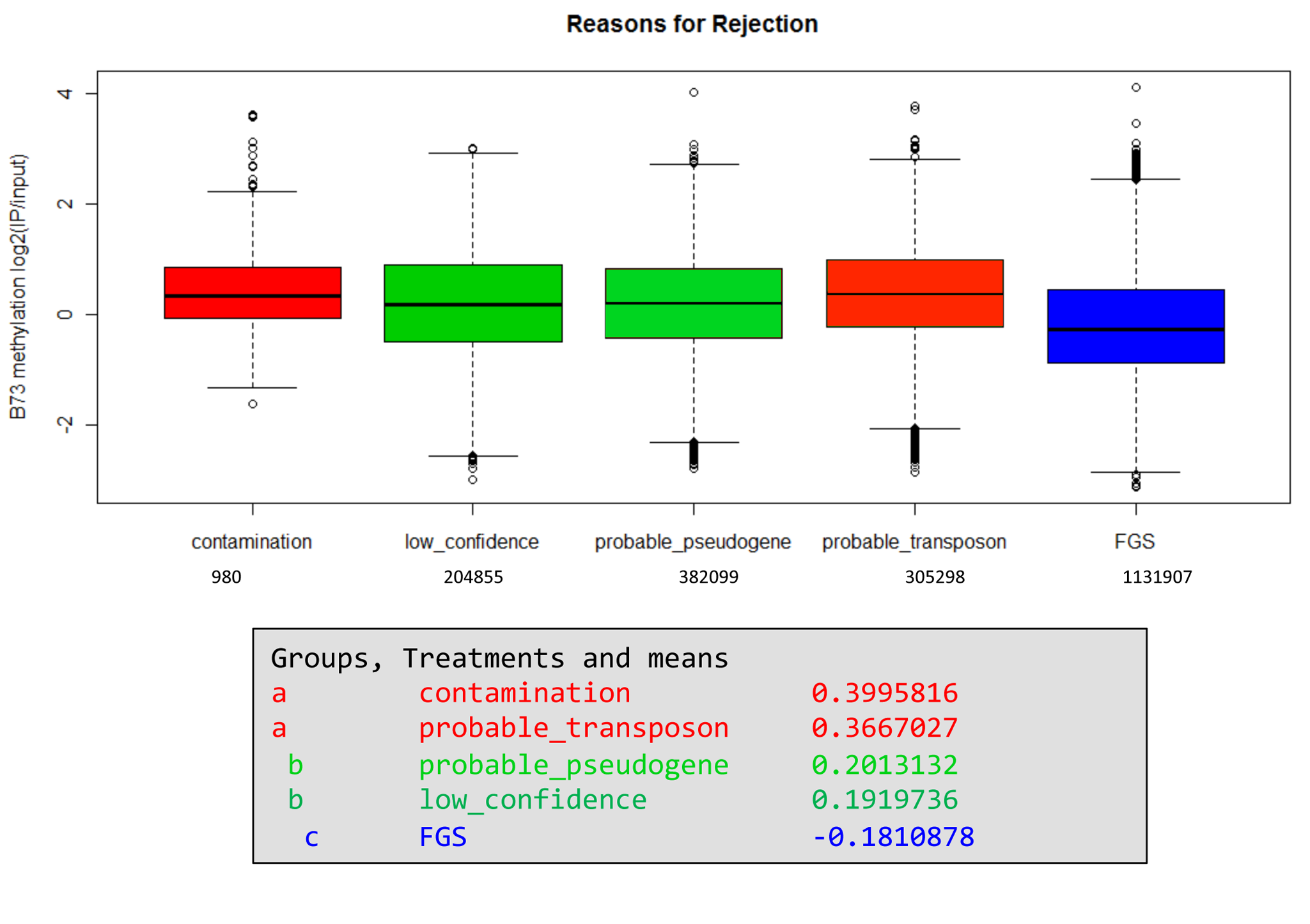

Supplement: Figure S5 — Increased methylation at rejected genes. The genes in the working set that were rejected from the FGS include possible contamination (bacterial sequences), low confidence FGENESH models, probable transposons and probable pseudogenes. Genes in each of these categories exhibit significantly higher methylation levels than genes in the FGS. (TIF) [file pgen.1002372.s005.tif]

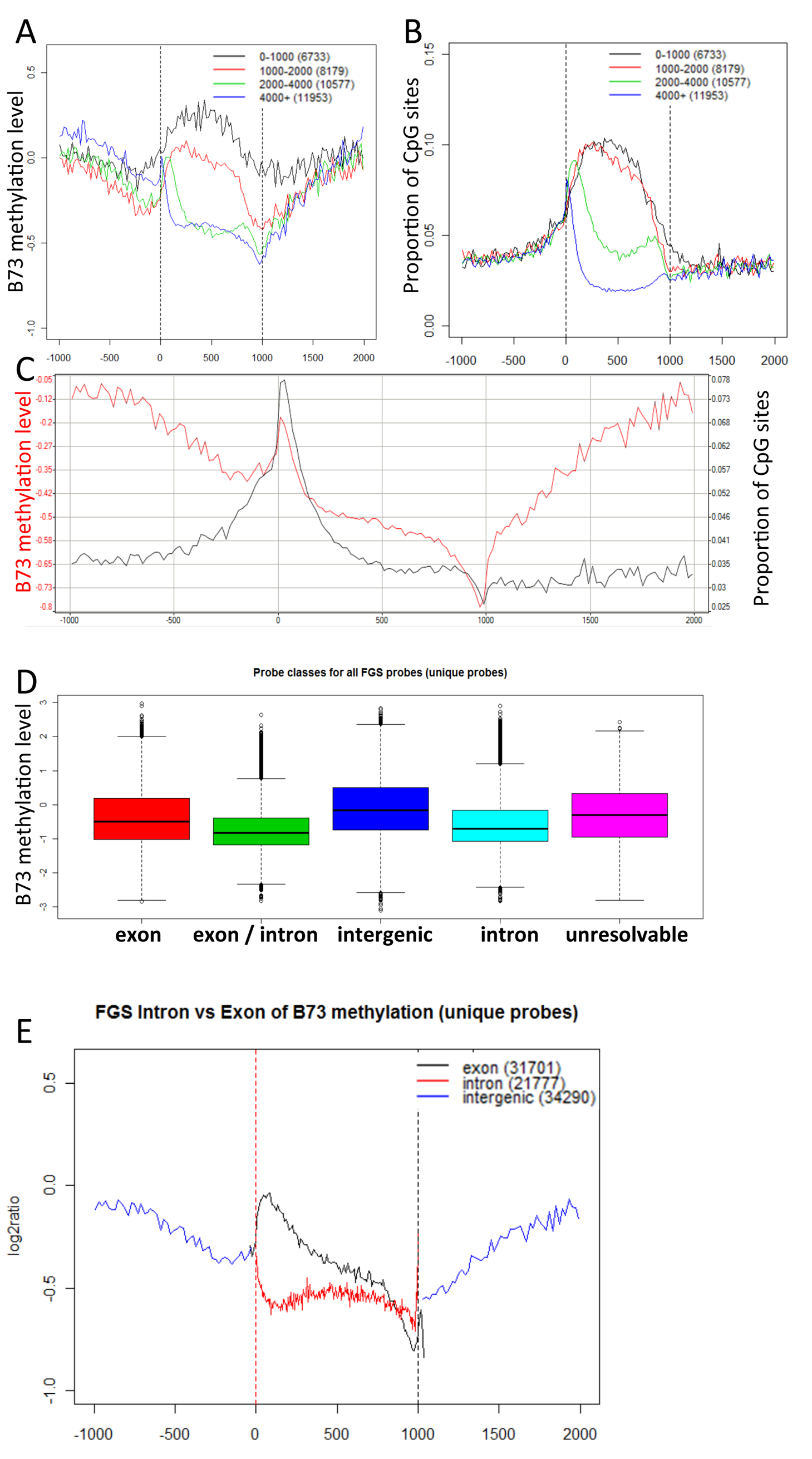

Supplement: Figure S6 — High levels of methylation within gene body. (A) The FGS genes were divided into different length categories to assess the level distribution of gene body methylation. (B) length categories also show increased CpG dinucleotide sites within the gene body. (C) Methylation levels and CpG dinucleotide proportions show related patterns within gene bodies. Methylation and CpG proportion diverge when not within genic sequence. (D) Methylation levels are higher in intergenic sequences than in exons and introns. The lowest levels of methylation are observed in introns and at exon/intron boundaries. (E) A profile of the methylation patterns along genes for only exon (black) or intron (red) shows that gene body methylation at the 5′ end of genes is confined to exons. Similarly, the reduced methylation at the 3′ end of genes is more pronounced in exons than in introns. (TIF) [file pgen.1002372.s006.tif]

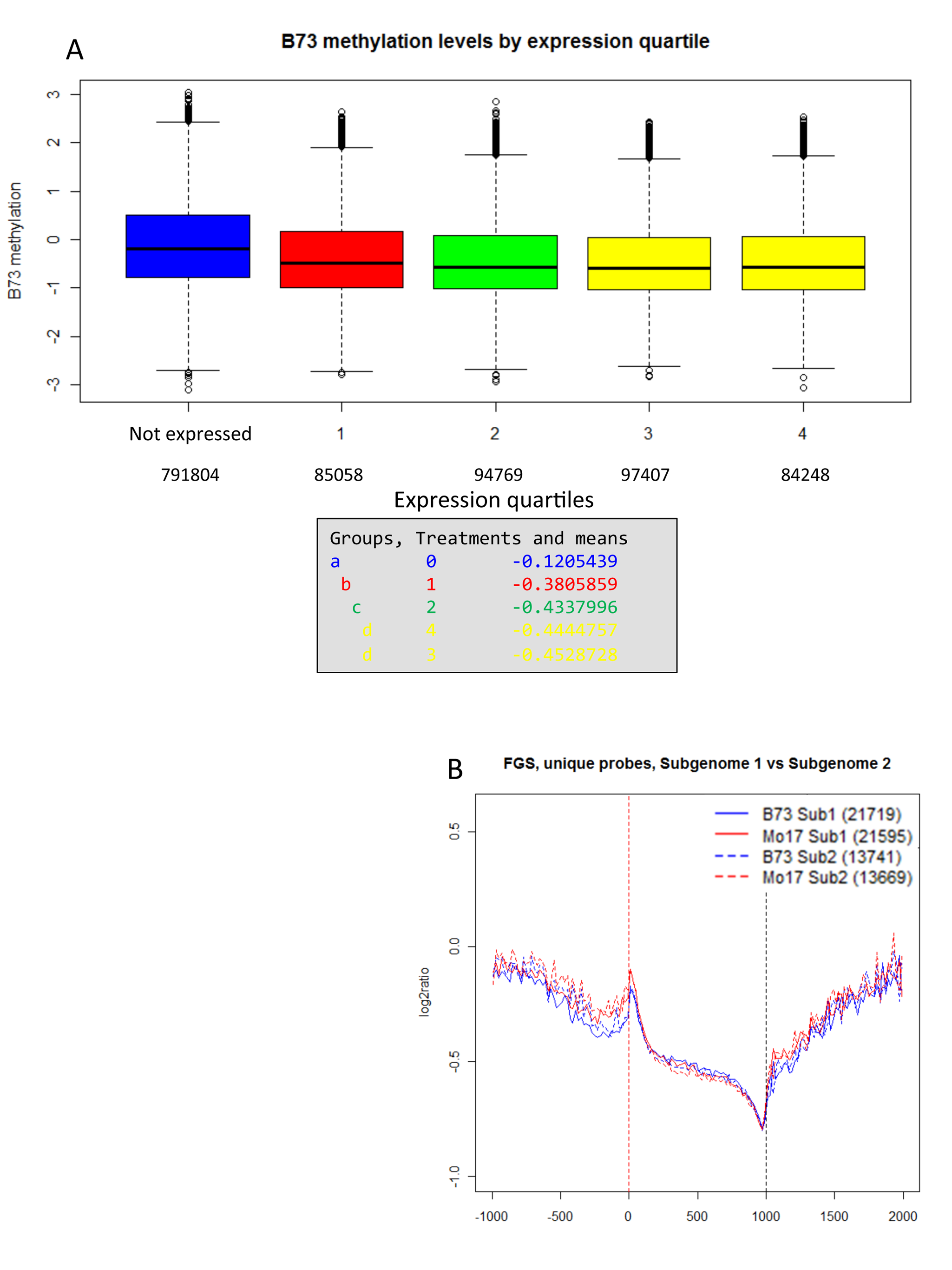

Supplement: Figure S7 — (A) Boxplot showing the different methylation levels between expression quartiles. Total number of probes in each category from the B73_unique probe set are presented under each category. Tukey HSD results are provided in gray box. (B) Methylation levels are not affected by sub-genome 1 and 2. The FGS genes were all classified based on whether they were located in regions of the maize genome classified as sub-genome 1 or sub-genome 2 (Based on [57]). There is no evidence for altered methylation levels for genes in sub-genome 1 relative to sub-genome 2. (TIF) [file pgen.1002372.s007.tif]

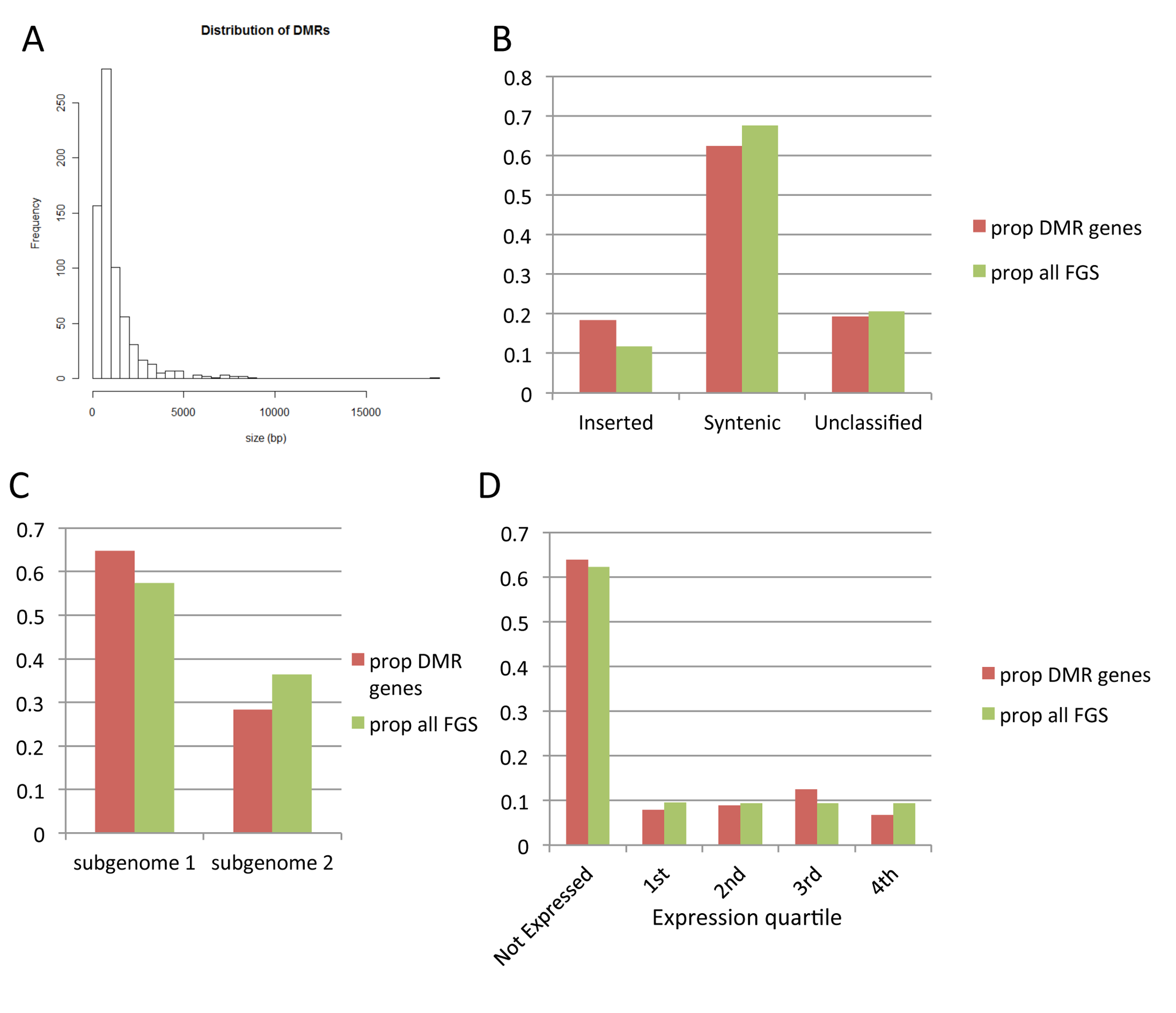

Supplement: Figure S8 — Characterization of maize DMRs. (A) A histogram is used to show the distribution of the length of the DMRs identified in B73 relative to Mo17. (B–D) The DMRs were analyzed to assess enrichments for syntenic positioning (B), subgenome classification (C), and expression quartile (D). For each comparison, the proportion of differentially methylated genes in each selected category were contrasted against the total number of genes in the filtered gene set. (TIF) [file pgen.1002372.s008.tif]
